# Comparison of Infection and Urosepsis Rates of Ciprofloxacin and Ceftriaxone Prophylaxis before Percutaneous Nephrolithotomy: A Prospective and Randomised Study

**DOI:** 10.1100/2012/916381

**Published:** 2012-12-17

**Authors:** Abdullah Demirtas, Yunus Emre Yildirim, Mustafa Sofikerim, Esma Gunduz Kaya, Emre Can Akinsal, Sevket Tolga Tombul, Oguz Ekmekcioglu, Ibrahim Gulmez

**Affiliations:** ^1^Department of Urology, Erciyes University Medical Faculty, 38039 Kayseri, Turkey; ^2^Department of Urology, Acıbadem University Medical Faculty, 34848 İstanbul, Turkey; ^3^Department of Microbiology, Erciyes University Medical Faculty, 38039 Kayseri, Turkey

## Abstract

This study aimed at determining the choice and administration duration of ideal antibiotic prophylaxis before percutaneous nephrolithotomy (PNL) operation, a treatment modality for nephrolithiasis. The study included 90 patients who had no internal problem, yet had a negative urine culture and underwent a PNL operation. We compared infection rates between ciprofloxacin and ceftriaxone groups and their subgroups. The results showed no statistical difference between ciprofloxacin and ceftriaxone groups in terms of systemic inflammatory response syndrome (SIRS) (CIP_*P*_ = 0.306,  CTX *P* = 0.334. As a result of this study no statistical difference was observed between ciprofloxacin and ceftriaxone in terms of SIRS. It seems, however, reasonable to choose ceftriaxone, considering antibiotic sensitivity of microorganisms and detection of three cases accepted as urosepsis in the ciprofloxacin group. As there is no difference between short, and long-term prophylactic use of these antibiotics, preference of short-term prophylaxis for patients with no risk of infection will be important to avoid inappropriate antibiotic usage.

## 1. Introduction

There are many treatment modalities of kidney stones. Due to high incidence and recurrence rates of the disease, removing stones from kidney should be ensured with minimal morbidity, maximal nephron preservation, and low recurrence rates, for successful treatment. Therefore, ESWL and minimal invasive surgical treatments are preferred rather than open surgical interventions. PNL does not cause any damage, or if does, only negligible damage to kidney. So it is accepted as first choice of minimal invasive treatment modality for patients requiring stone surgery. It is recommended that preoperative sterile urine should be provided with an appropriate antibiotic treatment for all patients. However, sepsis rates have been reported to vary from 0.25 to 1.5% in PNL studies [1–3].

In this study we aimed at detecting an appropriate prophylactic antibiotic and its duration before PNL, a treatment modality of nephrolithiasis.

## 2. Material and Methods

Ninety patients, were included to study PNL was indicated. Preoperative routine physical examination was performed in all patients. Complete blood count, blood urea nitrogen, creatinine, liver function test, urinalysis, and urine culture were investigated. Before surgery, stone size and location were determined by choosing at least one of following imaging methods: USG, IVU, or unenhanced CT. Patients with the following medical histories were excluded form study: UTI infections that would require antibiotic use within a year, antibiotic use within last month, immunosuppression treatment, internal problems that would affect SIRS criteria, and positive preoperative urine culture.

Patients were divided into two groups according to prophylactic antibiotic, being ciprofloxacin (CIPRO), and ceftriaxone (CTX) groups, before these two groups were divided into three subgroups. For the first subgroup only single dose of antibiotics was administered, rather than postoperative dose. The second subgroup was administered a preoperative single dose; the postoperative was discontinued following the one given in the 12th hour. For the 3rd subgroup, the daily dose antibiotic was continued after the first preoperative dose antibiotic and until nephrostomy tube was extracted. Daily dosages were arranged by renal capacity of the patients, as ciprofloxacin being 200 mg bid and ceftriaxone 1 gr bid. First two subgroups were evaluated as short term and 3rd group as long term. After standard premedication all patients were operated under intrathecal general anesthesia. Selected antibiotics were administered simultaneously with induction of anesthesia, or 30 minutes before induction.

Disinfection of surgical area was achieved with 10% povidone-iodine. All equipment used during operation was chosen from the same brand, and standard sterilization procedures were applied. After percutaneous access with needle was made, urine cultures from renal pelvis were collected from all patients. Removed stones were broken into small pieces and taken into sterile tubes for stone culture. Stones were kept in an oven at 36.5°C degree for a period of one day, for enrichment, and then inoculated to culture media.

Before removal of urethral and nephrostomy catheter postoperatively, both catheters were cleaned with 10% povidone-iodine and culture samples were taken from the most proximal part of them. Urethral catheters were removed on the postoperative 1st day. Nephrostomy tube was directly removed without clamping if vital sign was stable, urine from bladder and nephrostomy tube were clear, and there was no extravasation during anterograd pyelography and patient had no pain. Before removal, urine culture was taken form nephrostomy tube. On the other hand, patients with extravasations and bleeding were evaluated with anterograd pyelography following cleaning the urine form nephrostomy tube, usually within 2 or 3 days. Nephrostomy tube of SIRS-negative patients with extravasation and without pain was removed 12–24 hours after clamping. For SIRS-positive patients, broad spectrum antibiotic regimen for 7–14 days was initiated after this therapy nephrostomy catheter was removed. Bleeding, extravasation, infection, fever, thromboembolic events, urinoma, hematoma, and lung complications were recorded as an early postoperative complication.

In this study, we used SIRS criteria defined by the committee for consensus on the definition of sepsis, in order to define postoperative fever in a better way. Patients were followed postoperatively according to SIRS criteria: WBC < 4000 or >12000, heart rate >100 per minute, fever <36°C or >38°C, respiratory rate >20 per minute. Presence of two or more of these criteria was accepted as SIRS [4]. Presence of bacteruria or bacteremia with SIRS positive criteria was accepted as urosepsis in our study.

Postoperative first 48 hours patients were followed hourly. During postoperative follow-up fever was detected, routine urine and blood cultures were taken. Results of pre- and postoperative urine cultures, stone cultures, and blood cultures were evaluated for suitable antibiotic prophylaxis, infection, urosepsis, and ideal duration of prophylaxis.

## 3. Statistics

For statistical analysis of data from the study, SPSS (Statistical Package For Social Sciences) for Windows 15.0 was used. During evaluation of the data, in addition to descriptive statistical methods (mean, standard deviation), One-way ANOVA test and Student's *t*-test were used for comparison of numerical data and groups showing normal distribution. To compare group parameters without normal distribution, the Kruskal-Wallis test and Mann-Withney *U* test were used. Qualitative data was compared via Chi-Square test and Fisher's Exact Chi-Square test. For multiple comparisons, variant analysis was used. Results were assessed within 95% safety range and *P* < 0.05 statistical significance level.

## 4. Results

Mean age of patients was 43.9 ± 14.03 (20–83 years old) years old. 58 of them (64.4%) were male and 32 (35.6%) female. Mean stone burden was 5.1 cm² ranging from 0.7 to 2.7 cm². Before PNL operation, 5 (5.6%) patients had urinary diversion; 4 of them had nephrostomy and one had double J stent.

With preoperative imaging studies, which were US, CT, or IVU in 58 (64.4%) cases, no urinary obstruction was detected, while 32 (35.6%) cases demonstrated complete or partial urinary obstruction. Percutaneous access number per patient was single in 61 (67.8%) cases and two or more in 29 (32.2%) cases. Lithotripter was used in 70 (77.8%) patients. For the remaining 20 (22.2%) patients, it was not used. Mean surgery time was 85.7 minutes (15–330 min) and mean amount of 0.9% NaCl was 10.7 liters (2–60 liters) in our study. Hypotensive attack not requiring transfusion was observed in 4 (4.4%) patients while transfusion was executed in 15 (16.7%) patients due to perioperative bleeding. Also extravasation was detected on the postoperative 2nd or 3rd day, via anterograd pyelography in 5 (5.6%) patients. 11 (12.2%) patients were accepted as SIRS positive during postoperative period. No complication apart from the above-mentioned was detected. Mean duration of nephrostomy drainage was 3.2 days ranging from 2 to 10 days while all urethral catheters were removed on the postoperative 1st day.

In our study, patients (90 patients) were divided into two groups according to chosen prophylactic antibiotics: half of them (45 patients) in ciprofloxacin group and the other half (45 patient) in ceftriaxone group. Both groups were divided into three subgroups. Each subgroup included 15 patients. First two subgroups were named as short-term groups, and the last 3rd long-term group. Distribution of SIRS-positive patients among antibiotic groups is provided in Table 1.

These results show that there is no statistically significant difference between CIPRO and CTX group (*P* = 0.52). Additionally, there is no statistical difference between subgroups of both antibiotics (CIP *P* = 0.306 and CTX *P* = 0.334). Also long-term and short-term groups were not observed to be statistically different from each other (CIP *P* = 0.19 and CTX *P* = 0.59). 

Three SIRS-positive cases in the CIPRO group were accepted as urosepsis due to the postoperative bacteruria. *E. coli* was isolated from postoperative urine cultures of 2 urosepsis cases and *Enterococcus* species from the remaining one. Among these 3 cases with positive urine culture, one *E. coli* was sensitive CIPRO, while other *E. coli* and *Enterococcus* were resistant. On the other hand, in CTX group no bacteremia or bacteruria was detected in 4 SIRS-positive cases. However, for 5 of the 7 (71.4%) patients with SIRS (+) in the CIPRO group, their treatment regimen was converted into broad-spectrum antibiotics, due to suspicion of urosepsis and clinical instability, following a consultation of infectious diseases. In the CTX group, for 2 of 4 (50%) SIRS cases, the antibiotic regimens were changed due to clinical instability. Predictive parameters that would affect SIRS positivity among antibiotics are presented in Table 2.

In Table 3, the influence of culture positivity on SIRS in the antibiotic group is presented. According to these results, positive stone culture, renal pelvis urine culture, or postoperative urine culture are not statistically significant predictive factors that would affect SIRS positivity. There is no statistical difference between the numbers of positive cultures in antibiotic groups in terms of SIRS (*P* > 0.05), either. 

Multiple microorganisms diagnosed in 4 patients were isolated from their postoperative urine cultures. All of them are presented on Table 4. In our study, the most common culture positive sample was stone. Its culture positivity displayed an apparently higher rate than that of renal pelvis urine and postoperative urine. The most common isolated microorganism was coagulase negative *Staphylococcus* family, except S. saprofiticus, with a rate of 64.2%. Besides, *E. coli*, *S. saprofiticus*, and Proteus were isolated from stone cultures at rate of 21.4%, 3.5%, and 3.5%, respectively. Additionally, *E. coli* was isolated from 26% of the cultures. Mixed microorganisms were isolated from 4 of 11 patients with positive postoperative urine culture. Microorganisms isolated from these samples are presented at Table 4.

## 5. Comment

Percutaneous nephrolithotomy, as is known, is a commonly used surgical procedure for nephrolithiasis. Despite use of a prophylactic antibiotic, postoperative bacteremia and fever are reported as 37% and 74% [5]. In a surgical practice, despite application of prophylaxis and preoperative sterile urine, life-threatening systemic infections may develop [6]. No proved use of prophylactic antibiotic with preoperative sterile urine exists; however, short-term prophylaxis is generally preferred. This short-term prophylaxis is usually maintained up to the postoperative 48th hour [7]. Previous studies show that preoperative single antibiotic dose will be enough for patients with preoperative sterile urine culture, without a risk of upper urinary tract infection. In our study, one can state that there is no statistical difference between CTX and CIPRO either for single dose or long-term use. 

The study of Charton et al. sparked a critical argument about prophylaxis before PNL. That study stated that no major septic complication was observed without prophylaxis. Only 10% of the patients were exposed to fever and 35% of them suffered from urinary tract infection but they said that short-term prophylaxis was more reasonable [8]. Another study by Mariappan et al. alleged that ciprofloxacin prophylaxis administered for one week before PNL significantly decreased the urosepsis risk in 98 patients with stones >20 mm and/or pelvicaliceal dilatation that displays a fourfold risk of increase. That study 18 of 46 (39%) patients in the control group developed SIRS, whereas only 7 of 52 (13.4%) patients in the treatment group developed SIRS. They also reported that one-week administration of ciprofloxacin prophylaxis decreased positivity of pelvicaliceal culture three times, stone culture positivity two times, and risk of developing SIRS three times. That study indicated that 2 patients developing septic shock were diagnosed with positive stone culture, while 4 of 7 patients developing SIRS in the treatment group with infected stone culture, and one of them had positive pelvicaliceal culture [6]. 

As we know, SIRS is an important predictive factor for urosepsis. Prevalence of bacteruria or bacteremia with SIRS is accepted as urosepsis in our study. SIRS incidence after PNL is reported as 15.5% for the ciprofloxacin group and 8.8% for the ceftriaxone group. An important result concluded from our study is that 3 patients accepted as urosepsis are from ciprofloxacin group in which SIRS is seen to be relatively high. In the study arranged by Mariappan et al. that included 54 patients, while 42% of patients had positive upper urinary tract culture (pelvic and stone), only 5.6% of them had positive urine culture from bladder. There was no identical colonization between upper and lower urinary tract in their study [9]. In our study preoperative urine culture was sterile for all patients. 33.3% of patients had positive stone culture and pelvicaliceal culture. It is seen from our study that urine from bladder does not reflect all urinary system.

The incidence of infected stone culture is defined as 5.6–77.3% [10–14]. According to Gault et al. low incidence of infected stone (5.6%) in their study depends on long-term preoperative treatment with fluoroquinolones and lower prevalence of infected stone in Canada [10]. Fowler reported a 77.3% incidence rate and found that only 12.5% of patients with infected stone had positive urine culture form bladder [11]. Similarly, McCartnaey and Bratell repeated the weak correlation between infected stone and urine culture from bladder [12, 13]. Dogan et al. evaluated the data retrieved from 338 patients who underwent PNL retrospectively. They reported positive stone culture to be 34% (115/338) [15]. Similarly in our study, positive stone culture rate is 31.1% although all patients had sterile urine preoperatively. Our study and other studies emphasize that preoperative urine culture is not a predictive factor for postoperative stone culture.

One of the interesting results of our study is that pelvicaliceal urine culture is not enough to detect positive stone culture. Only one out of 28 patients with positive stone culture had positive pelvicaliceal urine culture. Dogan et al. reported similar results. They diagnosed only one patient with positive pelvicaliceal urine culture out of 19 patients with positive stone culture [16].

In our study group there was no colonization from blood culture of 3 urosepsis cases and other SIRS cases. Martin et al. observed bacteremia in only 50% of septic patients [17]. Again, Clayman defined the post-PNL bacteremia rate as 2% [18].

According to the study of Margel et al., positivity of stone or urine culture is accepted as relative risk for SIRS. They said that bladder urine culture is not enough to show an infection of upper urinary tract. In contrast, pelvicaliceal and stone culture are more powerful predictive factors for urosepsis. They also reported that bigger stones are more likely to get infected than smaller ones [19]. Again in the same study, it is said that patients with history of urinary tract infection are exposed to a higher risk of developing SIRS [19]. When Mariappan et al. compared different culture samples and SIRS, they found out that positive pelvicaliceal urine culture and stone culture increased risk of developing SIRS for four times [9].

Shigeta et al. pointed out to a critical conclusion in their study which included 54 patients. They diagnosed 10% of the patients with an infected stone and found out that in cases of a stone size bigger than 30 mm, bacteruria is more common [20]. In our study stone burden is related with SIRS or with urosepsis in the ciprofloxacin group but there is no such relationship with the ceftriaxone group. Similarly, Mariappan's study shows that stones are bigger in the cases developing SIRS. On the other hand, in cases without SIRS, infected stones are relatively smaller than the noninfected stones (29,8 mm–37,6 mm) [6]. In our study stone burden in cases with SIRS and positive stone culture is 4.4 cm^2^, while it is 4.3 cm^2^ in the group with negative stone culture.

Importance of preoperative factors, such as infected stones, positive cultures, more stone burden, longer operation time, more irrigation fluid, and participating the systemic infections are reported in numerous articles [9]. In our study, however, only operation time was found to be related with SIRS in both antibiotic groups.

Postoperative fever will not be important if patients given antibiotic pre- and postoperatively have negative urine culture, and if hemodynamically they are stabile because postoperative fever does not always mean sepsis. With regards to this issue, Cadeddu et al. stated that an urgent investigation of postoperative fever is unnecessary if preoperative sterile culture is prevalent, preoperative antibiotic is given and patient is hemodynamically stable [21]. To our knowledge, there is no need to initiate or change antibiotics for hemodynamically stable patients with postoperative fever. But thorough management of septic complication together with a microbiologic investigation should be done with cultures during preoperative, postoperative, and febrile periods. 

In our study colonization of microorganisms was not documented in every patient with postoperative fever or SIRS. Possibly, this may be because prophylactic antibiotic prevents proliferation of microorganisms, or increased inflammatory mediators trigger postoperative fever by surgical manipulation. We assume that the rates of infection and urosepsis in our series are lower than those reported in the literature, due to selection of patients with no internal problem, no risk of urinary tract infection, yet who have a normal urinary structure, or use of effective prophylactic antibiotics against microorganisms that would lead to contamination. 

Truck and Bronsema mostly isolated *E. coli* and usually other microorganisms like Proteus, Klebsiella, *Enterobacter* and *Pseudomonas*, from population after PNL, during endoscopic procedures in their studies [22, 23]. In our study, the most commonly isolated microorganisms from all culture samples are coagulase negative *Staphylococcus*, and the second one is *E. coli* with a rate of 26%. Related to this, Margel et al. isolated *Enterococcus*, a gram-positive bacterium with rate of 74% [19]. In our study coagulase negative *Staphylococcus* is the most commonly isolated organism from positive stone culture (64%). In the multicentre study performed by Farrell et al., ciprofloxacin was found to be quite effective against uropathogenic microorganisms. In this study antibiotic sensitivity rates of *E. coli*, *Klebsiella pneumonia*, *Pseudomonas auroginosa*, and Proteus mirabilis are, respectively, reported to be 98%, 94%, 89% and 87% [24]. Although we did not perform antibiotic sensitivity for all positive cultures, and gram-positive organisms were isolated usually from culture samples, isolated gram-positive and negative organisms seemed to have similar sensitivity for both ciprofloxacin and ceftriaxone. 

## 6. Conclusion

The number of randomized studies is not adequate to compare the infection rates between antibiotic groups after endoscopic surgeries like PNL. For this reason, it is still controversial as to which antibiotic should be given with what duration during surgeries, such as PNL, in which the urinary tract is opened. Absence of certain algorithms about prophylaxis and presence of different regimens applied by clinics result in a developed antibiotic resistance.

## Figures and Tables

**Table 1:** Number of SIRS cases among antibiotic groups.

	Ciprofloxacin (CIPRO)				Ceftriaxone (CTX)			
	1st Group	2nd Group	3rd Group	Total (*N*)	1st Group	2nd Group	3rd Group	Total (*N*)
SIRS (+)	1 (2.2%)	2 (4.4%)	4 (8.8%)	7 (15.5%)	2 (4.4%)	—	2 (4.4%)	4 (8.8%)
SIRS (−)	14 (31.1%)	13 (28.8%)	11 (24.4%)	38 (84.4%)	13 (28.8%)	15 (33.3%)	13 (28.8%)	41 (91.1%)

Total (*N*)	15 (33.3%)	15 (33.3%)	15 (33.3%)	45 (100%)	15 (33.3%)	15 (33.3%)	15 (33.3%)	45 (100%)

*No significant difference between ciprofloxacin and ceftriaxone in terms of SIRS (*P* = 0.52).

**No statistically significant difference between subgroups of both antibiotics in terms of SIRS positivity (CIP *P* = 0.306, CTX *P* = 0.334).

*** No significant difference between short- and long-term groups of both ciprofloxacin and ceftriaxone in terms of SIRS (CIP *P* = 0.19, CTX *P* = 0.59).

****Chi-Square test.

**Table 2:** Comparison of the predictive factors affecting SIRS positivity among antibiotic groups.

	CIPRO			CTX		
	SIRS			SIRS		
	(+)	(−)	*P* value	(+)	(−)	*P* value
Stone burden (cm^2^)	5.2	4.2	0.032	4.1	5.9	0.873
Operation duration (min)	124	72	0.025	112	89	0.115
Amount of NaCl (liter)	12	9.4	0.220	15	11	0.297
Duration of nephrostomy (day)	5.8	3.1	0.003	4.5	2.7	0.009
Multiple access	71.4%	26.3%	0.032	25%	31.7%	1.000
Presence of obstruction	42.9%	44.7%	1.000	25%	26.8%	1.000
Lithotripter use	100%	71.7%	0.168	50%	82.9%	0.173

*Mann-Whitney *U* test.

**Table 3:** Effect of culture positivity on SIRS between CIPRO and CTX groups.

	CIPRO			CTX		
	SIRS			SIRS		
	(+)	(−)	*P* value	(+)	(−)	*P* value
(+) Stone culture (%)	14.3%	26.3%	0.663	25%	39%	1.000
(+) Renal pelvis urine culture (%)	14.3%	2.6%	0.290	0	2.4%	1.000
(+) Postop. urine culture (%)	42.9%	13.9%	0.094	0	7.3%	1.000

*No statistically significant difference was observed in positive cultures between ceftriaxone and cetrifiaxone in terms of SIRS positivity. (*P* > 0.05).

**Chi-Square test.

**Table 4:** Distribution of isolated microorganisms between antibiotic groups.

	Pelvic system culture		Stone culture		Postop. urine culture	
	CIPRO	CTX	CIPRO	CTX	CIPRO	CTX
*E. coli *	2	—	1	5	3	1
Coagulase (−) *Staphylococcus *	—	—	8	10	3	1
*Pseudomonas *	—	—	—	2	—	—
*Enterococcus *	—	—	—	—	2	1
*Acinetobacter *	—	—	—	—	1	—
*S. saprofiticus *	—	1	1	—	—	—
Proteus	—	—	1	—	—	—
*Citrobacter *	—	—	—	—	1	—
Others	—	—	—	—	2	—
